# A Metagenomic Approach to Evaluating Surface Water Quality in Haiti

**DOI:** 10.3390/ijerph15102211

**Published:** 2018-10-10

**Authors:** Monika A. Roy, Jean M. Arnaud, Paul M. Jasmin, Steve Hamner, Nur A. Hasan, Rita R. Colwell, Timothy E. Ford

**Affiliations:** 1 Department of Environmental Health Sciences, School of Public Health & Health Sciences, University of Massachusetts Amherst, Amherst, MA 01003, USA; monikaroy@umass.edu (M.A.R.); jarnaud@umass.edu (J.M.A.); shamner@montana.edu (S.H.); 2 Biotechnology Training Program, University of Massachusetts Amherst, Amherst, MA 01003, USA; 3 Equipes mobiles d’intervention rapide (EMIRA) du Ministère de la Santé Publique et de la Population (MSPP), Hinche HT 5111, Haiti; drpjasmin@gmail.com; 4 CosmosID Inc., 1600 East Gude Drive, Rockville, MD 20850, USA; nur.hasan@cosmosid.com (N.A.H.); rcolwell@umiacs.umd.edu (R.R.C.); 5 Center for Bioinformatics and Computational Biology, University of Maryland, College Park, MD 20742, USA

**Keywords:** cholera, metagenomic analysis, bioinformatics, whole genome sequencing, principle components analysis, water quality, environmental sampling, Haiti

## Abstract

The cholera epidemic that occurred in Haiti post-earthquake in 2010 has resulted in over 9000 deaths during the past eight years. Currently, morbidity and mortality rates for cholera have declined, but cholera cases still occur on a daily basis. One continuing issue is an inability to accurately predict and identify when cholera outbreaks might occur. To explore this surveillance gap, a metagenomic approach employing environmental samples was taken. In this study, surface water samples were collected at two time points from several sites near the original epicenter of the cholera outbreak in the Central Plateau of Haiti. These samples underwent whole genome sequencing and subsequent metagenomic analysis to characterize the microbial community of bacteria, fungi, protists, and viruses, and to identify antibiotic resistance and virulence associated genes. Replicates from sites were analyzed by principle components analysis, and distinct genomic profiles were obtained for each site. Cholera toxin converting phage was detected at one site, and Shiga toxin converting phages at several sites. Members of the *Acinetobacter* family were frequently detected in samples, including members implicated in waterborne diseases. These results indicate a metagenomic approach to evaluating water samples can be useful for source tracking and the surveillance of pathogens such as *Vibrio cholerae* over time, as well as for monitoring virulence factors such as cholera toxin.

## 1. Introduction

The emergence of cholera in Haiti, following the severe (7.0 Richter scale) earthquake south of Port-au-Prince in 2010, resulted in 817,000 cases of illness and more than 9749 deaths through 2016, according to the Haitian Ministry of Public Health and Population (MSPP) [1]. As a result of a coordinated response between the MSPP, the U.S. Centers for Disease Control and Prevention (CDC), the United Nations Children’s Fund (UNICEF), the Pan American Health Organization (PAHO), the World Health Organization (WHO), and other local and international health partners, external financial resources were raised to strengthen Haiti’s disease surveillance and response systems. While these efforts made tremendous strides in the country, the lack of continual funding for surveillance activities threatens progress towards eliminating diseases like cholera [2].

Cholera infection is caused by the bacterium *Vibrio cholerae* and is characterized by acute diarrhea and vomiting; the illness can range from subtle to severe, leading to death [3]. Cholera is a waterborne disease, with poor water and sanitation infrastructure a primary risk factor for transmission [4]. A significant correlation has also been found between rainfall and cholera incidence in Haiti, with a 4–7-day lag time [5]. Cholera has declined since its initial outbreak, with a total of 13,681 cases and 159 deaths in 2017 [1]. However, with declining surveillance funding, continued poor water quality, failing infrastructure, and future hurricane risks, a need exists for reliable and low-cost diagnostics for improving surveillance, prediction, and response to outbreaks using measurable environmental parameters [6,7].

Diagnostics tools for environmental sampling are being tested to shorten the time and reduce the number of expert personnel needed to determine cholera presence in a limited resource setting [8]. Several rapid diagnostic tests (RDTs) have been developed for cholera detection including the Crystal VC Dipstick, Artron *V. cholerae* O1 and O139 Combo Test, and the SD Bioline Cholera Ag O1/O139 RDT. These tests have significant performance variation, suggesting that they may be useful for initial surveillance in low resource settings, but that improvements are needed prior to their use in clinical settings [9]. A relatively inexpensive membrane filtration plate count technique has also been developed and tested that accurately quantifies *V. cholerae* in a mixed-culture setting [10], however, this method requires 24 hours of incubation time in specific laboratory settings. 

PCR methods have provided useful information for detecting cholera at specific locations post-outbreak [11,12,13]. However, whole genome shotgun sequencing combined with advanced bioinformatic analysis offers a method to examine the genetic diversity of environmental samples and to characterize sites in terms of relative abundance of bacteria, fungi, protists, viruses, antimicrobial resistance (AMR), and virulence associated genes [14]. Metagenomic approaches are now being used to assess and characterize the microbiota and bacterial communities in diverse waters, ranging from Amazonian rivers to water sources in urban environments [15,16,17]. This method provides a way to not only identify factors related to cholera presence, but also provide characterization of factors related to other waterborne diseases present in Haiti. 

To our knowledge, only limited environmental monitoring of surface waters is currently conducted in Haiti. The University of Florida has established environmental monitoring sites in the Gressier/Leogane region of Haiti, west of Port-au-Prince, but the focus of sampling is only on monitoring environmental reservoirs of toxigenic *V. cholerae* 01 [11,13]. In the Artibonite region of the Central Plateau, focus has also been on the isolation of *V. cholerae*, where basic water quality parameters have been collected from the Artibonite River and tributaries. The results suggest high levels of *E. coli*, with a geometric mean of 500 CFU/100 mL (this included some samples taken from rivers outside the Artibonite region), with 74% of samples considered in the high risk category according to WHO guidelines [18].

Continuous water quality monitoring is needed in Haiti for public health purposes in assessing the epidemiology of waterborne diseases like cholera [19]. While still used on a point sampling basis, the use of metagenomics provides a powerful tool that yields more profiling information beyond *V. cholerae* presence, and has the potential to be used in a clinical setting. At present, hand-held sequencing technologies, such as the Oxford Nanopore Technologies handheld MinION, are expensive, at around $1000 per sample for reagents and a single flow cell. However, the potential for multiplexing up to 12 samples could bring the cost down to approximately $80 per sample [20]. Advances in barcoding chemistry [21], and the purchase of flow cells in bulk, also have the potential to further reduce per sample costs. 

Although bioinformatics technologies are improving, further optimization is necessary before routine diagnostics for field use are possible. However, the advances made thus far make this technology comparable to the cost of bench-top instruments, but in a handheld, mobile device. With the development of handheld DNA sequencing technologies that make such sample analysis in remote locations and in as little as a few hours possible, this type of sample collection and metagenomic analysis could soon be conducted on a local scale in Haiti [22]. This paper explores the utility of using a metagenomic approach to evaluate surface water quality, and provides data on the relative abundance of pathogens from several sites in Haiti. 

## 2. Materials and Methods

### 2.1. Water Sampling and Shipping

**Figure 1:**
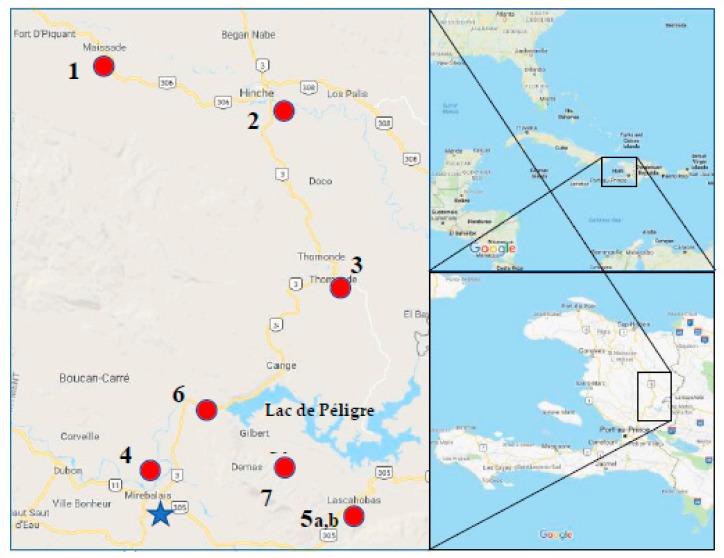
Sampling sites in the Central Plateau of Haiti (Mapdata©2018 Google). A blue star denotes the first cluster of cholera cases just south of Mirebalais [23]. Sites labelled 1 through 5a were sampled in triplicate in January 2018. Single samples were taken from sites labelled 6 and 7 in July 2017. Sites 1, 4 and 5b were also sampled in July 2017.

Triplicate water samples were collected between 5–6 January 2018 from five surface water sites in the Central Plateau region of Haiti: Maïssade, Hinche, Thomonde, Mirebalais, and Lascahobas (Figure 1, Table 1). Information on elevation, average temperature, and average rainfall for January and July at largely populated communities close to these sampling sites is provided in Table 2. This reflects the significant seasonal differences in rainfall between these sampling dates. These sites were chosen based on ease of sampling, while at the same time targeting areas with the highest level of cholera incidence as reported by the MSPP. These sites are close to river crossings on roads linking the major towns in the Central Plateau region, including the Rivière La Thème in Mirebalais, close to the first reported cholera cases in 2010. Due to their proximity to river crossings, these sites are heavily used by communities for bathing, washing clothes and dishes, and other household activities where water could be inadvertently consumed. This may be a particular risk for young children and infants. Samples had also been collected the previous summer, between 24–28 July 2017, but were not replicated or collected from the exact same locations, though they were collected from the same region. They are included to provide a seasonal comparison. 

At each of the five sites for the January 2018 sampling, 250 mL water samples were collected and filtered directly on-site in triplicate using Sterivex^TM^ filters (0.22 µm pore size, polyethersulfone sterile membranes; Millipore^®^ Sigma, St. Louis, MO, USA). These samples were collected approximately 10 m apart to minimize sediment disturbance. Samples were placed on ice and transported directly to Port-au-Prince for shipping to the U.S. The shipping company, Deutsche Post DHL Group (DHL), required a letter from a Haitian organization before shipping the samples. This caused extensive delays until a letter was obtained from a local partner organization, Midwives for Haiti (MFH), based in Hinche, Haiti on 17 January 2018. The samples remained frozen during this waiting period. Regrettably, the samples were unable to be shipped with ice blocks. They arrived at CosmosID in a thawed state six days later, when they were immediately frozen prior to downstream processing and analysis. For samples collected the previous summer in July 2017, several ~100 mL samples were collected at a selection of sites, and these samples were transported over a 2-day period at room temperature to the U.S., whereby they were immediately frozen prior to downstream processing and analysis.

### 2.2. Metagenomic Sequencing and Bioinformatics Analysis

Metagenomic DNA was extracted from the filters using a DNeasy PowerWater Sterivex Kit (QIAGEN) following the manufacturer’s guidelines. Concentrations of the metagenomic DNA were measured using a Qubit Fluorometric Quantitation (Thermo Fisher Scientific, Waltham, MA, USA). Most samples yielded around 1–4 ng/μL genomic DNA (Table S1). Fragment libraries were constructed from 100 ng DNA (except for one sample with low yield where 15 ng DNA was used) using the Thermo Fisher IonXpress Plus Fragment Library kit (Thermo Fisher Scientific) according to the recommended manufacturer instructions. Genomic DNA libraries were quantified by qPCR and then sequenced on an Ion S5 XL Semiconductor Sequencer (Ion Torrent, Thermo Fisher Scientific) to generate 200 bp sequence reads. Each sample was sequenced with an average of 17M sequence read depth. General Sequencing Statistics of all samples, as well as Mean Sequence Quality distribution as measured by MultiQC [25] are illustrated by Figures S1 and S2, and Table S1. As the mean quality value across each base position in the read was always above quality score 17 for at least 80% of the read length (i.e., probability of correct base call ~98%), reads were not subjected to quality trimming. Raw genomic sequences were analyzed by CosmosID metagenomic software [26,27,28,29,30,31], including principle components analysis (PCA), to reveal microbial community composition, antibiotic resistance markers, and virulence gene pools. 

Briefly, the CosmosID platform utilizes high performance data mining algorithms and highly curated dynamic comparator databases (GenBook^®^) that rapidly disambiguate hundreds of millions of short reads of a metagenomic sequence into the discrete microbial genomes and genes engendering the identified sequences without the need for sequence assembly. Similarly, the community resistome and virulome, and the collection of antibiotic resistance and virulence associated genes, respectively, in the microbiome were also identified by querying the unassembled sequence reads against the CosmosID curated antibiotic resistance and virulence associated gene database. The GeneBook database is composed of over 150,000 microbial genomes and gene sequences representing bacterial, viruses, protists, and fungi, as well as antibiotic resistant and virulence associated genes. The curated databases provide extremely fine resolution in identification, discrimination of pathogens from ‘near neighbors’, and accurate measurement of relative abundance. Results are either reported as “filtered”—which is based on internal statistical scores that indicate the organism or gene is most likely present, or “unfiltered”—where further validation is recommended to confirm their presence (CosmosID documentation https://app.cosmosid.com/docs). Data are deposited in the NCBI Sequence Read Archive (SRA) database with accession number SRP158812. The resultant taxa abundance tables were used to calculate observed species richness, alpha diversity indices, and beta diversity distance matrices (data not shown for beta diversity). PCA was performed to cluster samples based on abundance using the covariance matrix of normalized data as the measure of similarity.

## 3. Results

### 3.1. Environmental Sampling

Sampling sites were selected as previously described, and are depicted in Figure 1. Sampling sites from January 2018 are labelled 1 through 5a, and sampling sites from the previous summer in July 2017 are labelled 1, 3, 4, 5b, 6, and 7. The location and description of each site are described in Table 1. Syringe cartridges were used to collect water samples to filter through Sterivex filters at all sites during both time points. 

### 3.2. Sequencing Analysis

**Figure 2:**
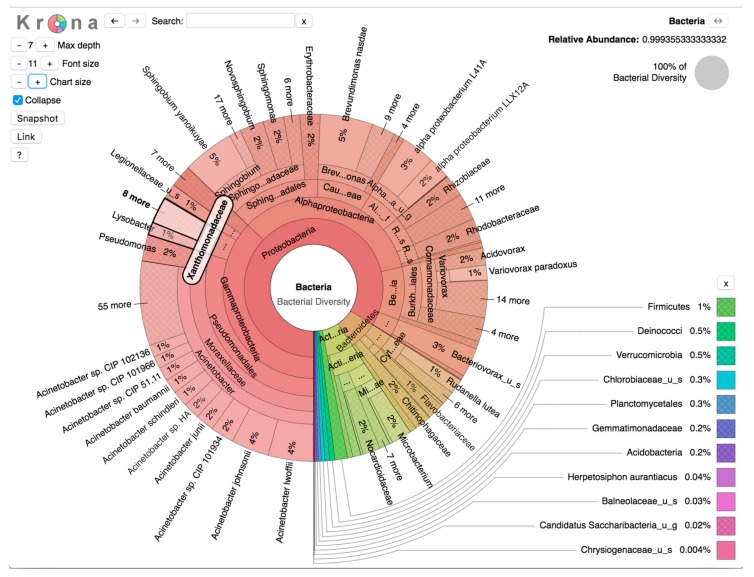
Krona visualization. Total bacterial diversity, representing gamma diversity, among all samples from the January 2018 time point.

Sequence analysis of DNA extracted from the samples revealed a wide diversity of bacteria, with over a thousand strains of sequences at each site. Figure 2 depicts a Krona [32] visualization of all bacteria detected in January 2018 samples across the five sites. The predominant phylum of bacteria was the gram-negative Proteobacteria at 84% of total bacterial diversity. This phylum includes a wide variety of pathogens, and in this analysis Alphaproteobacteria comprised 41%, Betaproteobacteria 11%, and Gammaproteobacteria 44% of Proteobacteria. Within the Gammaproteobacteria class, the Pseudomonadales order comprised 81% (30% of total bacterial diversity), of which 92% was of the *Acinetobacter* genus (27% of total bacterial diversity). The Gammaproteobacteria class also includes *V. cholerae* in the Vibrionales order.

**Figure 3:**
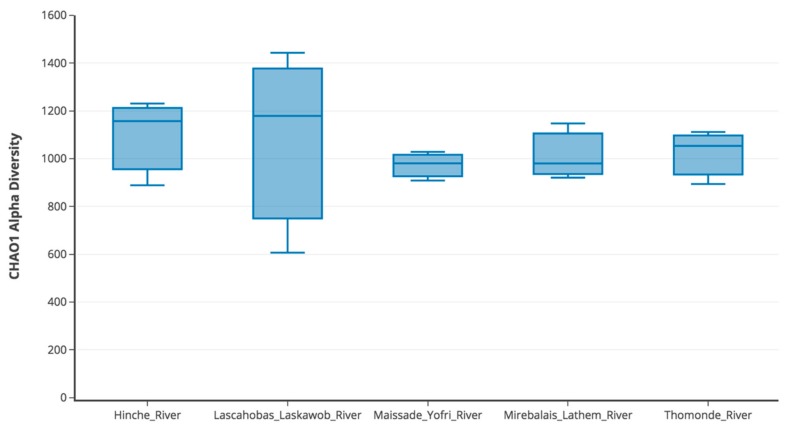
Choa1 alpha diversity. All three replicates of each site are represented by a box plot. Raw data used to generate the box plots are presented in Supplemental Table S2.

Among the five sampling sites from the January 2018 time point, mean species diversity was calculated to represent alpha diversity (Figure 3). All sites demonstrated similar levels of diversity, however, replicates from the Lascahobas River site showed the most variability. 

**Figure 4:**
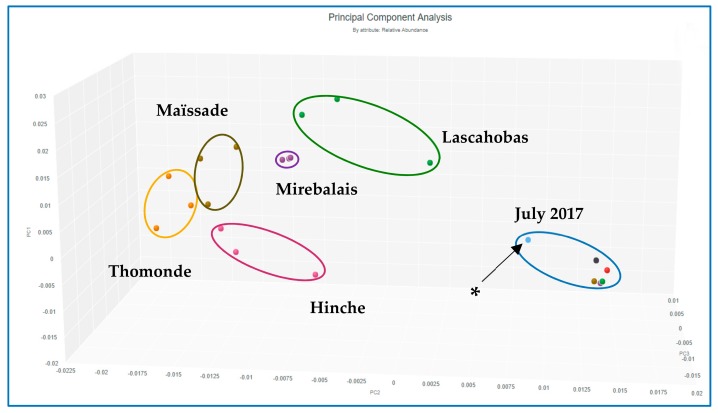
Principle components analysis (PCA) of relative abundance of bacteria at all sites, including July 2017 samples, which were not replicated (* Site ~1 mile below Lac de Péligre dam).

**Figure 5:**
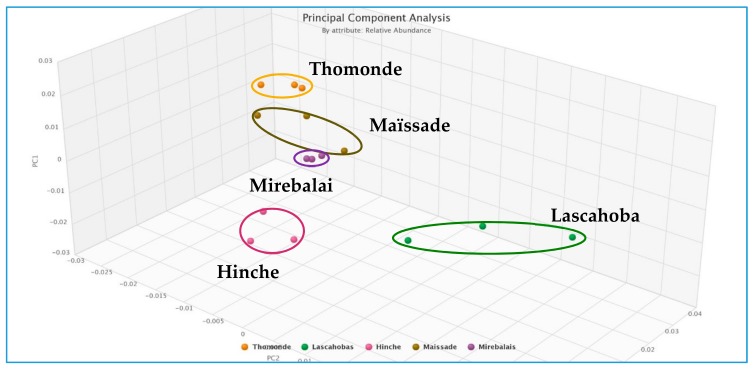
PCA of relative abundance of bacteria from replicate samples collected in January 2018.

Each site also contained a relatively unique bacterial composition, or fingerprint, demonstrated by PCA performed on data gathered for all sites across the two sampling time points (Figure 4), and for the January 2018 sites alone (Figure 5). Supplemental Figure S3 shows dominant bacteria identified for all sites in the January 2018 sampling. Several *Acinetobacter* spp. appear in all sites, particularly the Lascahobas site where they comprise 10 of the top 12 bacterial genera detected in terms of relative abundance. In contrast, the other 4 sites had more bacterial diversity and *Acinetobacter* spp. comprised about 5 of the top 11–13 bacteria detected. 

The relative abundance of the *E. coli* and *V. cholerae* bacteria, the *V. cholerae Intl1* virulence gene, and the *Stx2*-converting phage was quantified across replicates for each of the five sites in the January 2018 sampling period, and for each site in the July 2017 sampling period. These data are presented in Table 3. For both January 2018 and July 2017 sampling, *E. coli* was present in all replicates, although generally at low abundance. *V. cholerae Intl1* was present in most replicates but with a wider range of abundance; of note, the relative abundance of *Intl1* in Maïssade samples in July 2017 was much higher at 17.65% than samples collected in January 2018, all below 5.0%. Replicates for Mirebalais were all consistently higher for relative abundance of *Intl1*, above 7.0%, but one replicate exhibited much higher abundance at 18.45%. *V. cholerae* was detected in some replicates across all sites and sampling periods, but at very low relative abundance, with the highest detection level of 0.02%. *Stx2*-converting phage was also detected in some replicates across all sites, but ranging from not detected to 9.82% relative abundance. 

Supplemental Figures S4–S6 provide filtered sequence data on viruses, virulence factors and AMR genes for each site. Filtered data is used to allow these figures to be manageable. In the case of viruses, filtered data only detected viruses at 9 of the 15 sample sites, so a relative view is not possible (Figure S4). However, a large number of viruses were detected at lower confidence in the unfiltered data. Viruses were dominated by phages and associated with a wide range of potentially pathogenic bacteria. In the filtered sample set, Enterobacteria phages were dominant, with specific *Escherichia* and *Salmonella* phages present. In unfiltered data, additional phages were identified and associated with bacteria such as *Aeromonas*, *Acetobacter*, *Arthrobacter*, *Bacillus*, *Bordatella*, *Burkholdaria*, *Clostridium*, *Cronobacter*, *Haemophilus*, *Mycobacteria*, *Pseudomonas*, *Shigella*, *Staphylococcus*, *Streptococcus*, *Vibrio*, and *Yersinia*—all genera with important human pathogenic species. In addition, human mastadenovirus was detected. Due to the number of virulence factors detected, only the top factors representing 80% of total abundance are presented in Supplemental Figure S5. Virulence factors are dominated by those associated with the important pathogens, *Klebsiella pneumonia*, *Proteus mirabilis*, *Pseudomonas aeruginosa*, *V. cholerae*, and *E. coli*. Other pathogen-associated virulence factors are also present. Antimicrobial resistance genes are dominated by those conveying aminoglycoside, sulphonamide, beta-lactam, and in some cases tetracycline resistance (Figure S6).

Information on viruses, virulence factors, and AMR genes present in the most contaminated replicate of samples collected from the Thomonde site are described in Table 4. Notable is detection of *Stx2*-converting phage and *Vibrio* phage CTX. 

Supplemental Figures S7 and S8 present information on fungi and protists. Dominant fungi include *Onygenales* spp., *Epichloe sylvatica*, *Puccinia arachidis*, *Clavaria fumosa*, *Lentinus polychrous*, and at two of the Lascahobas sites *Candida parapsilosis* (1.92% and 2.39% abundance), *Enterocytozoon bieneusi* at one Hinche River site (2.52%) and two Lascahobas sites (1.98% and 2.85%), and *Anncaliia algerae* at two Hinche River sites (4.58% and 2.78%), one Mirebalais site (2.76%), and two Lascahobas sites (4.48% and 4.67%). Unfiltered results also identify *Candida albicans*, *Alspergillus fumigates*, and *Pneumocystis jirovecii*.

Protist sequences are dominated by *Paramecium biaurelia* and *Pseudoperonospora cubensis*. However, *Acanthamoeba polyphaga* is present at one Hinche and one Mirebelais site (13.37 and 30.72%, respectively). Also present at almost all sites and highest at one Hinche River site (4.15%) is *Plasmodium falciparum*. Unfiltered data (not shown) also suggests sequence evidence for *Entamoeba* spp., *Toxoplasma gondii*, and *Trypanosome congolense*.

## 4. Discussion

The data presented in this study provide relative abundances of bacteria, fungi, protists, and viruses, as well as identify antibiotic resistance and virulence associated genes for several sites in samples collected in Haiti over two time points, using a metagenomic approach.

### 4.1. Limitations

We are aware of the limitations of this study based on the failure of the cold chain due to the unanticipated refusal of the shipping company in Haiti to ship ice packs. For future studies, we are working with colleagues in Haiti for DNA extraction and preservation with DMSO-EDTA-salt (DESS) [33] prior to shipping. However, in this study the possibility of both growth and inhibition of select species call into question our measurements of relative abundance. In past work, members of the research team have had no problems recovering and isolating specific pathogens, including *V. cholerae* and enterohemorrhagic and enterotoxigenic *E. coli* (EHEC and EPEC) strains, from filters shipped wet from India [34]. So, even though our current work may not be quantitative, we believe the qualitative findings on specific pathogens are significant.

Despite these limitations, several interesting trends are worth noting. The Mirebalais Rivière La Thème site showed greatest consistency in replicates, as demonstrated by PCA (Figure 4 and Figure 5). This site is located near the epicenter of the 2010 cholera outbreak [23]. Other sites showed greater diversity among replicates, which may reflect the heterogeneity of the river samples collected at multi-use sites. Overall, PCA analysis was able to distinguish the five sites sampled in January 2018, and demonstrated that it was possible to characterize the sites in terms of bacterial community structure. This finding is helpful for source tracking of groups of pathogens and determining potential sources of contamination for future studies.

### 4.2. Seasonal Differences

As shown in Figure 4, there is a clear difference between results obtained in July 2017 and in January 2018, even though some samples were collected at the same sites. Samples collected in July 2017 demonstrated a different bacterial community composition compared to samples from any of the sites sampled in January 2018. This may be due to seasonal differences between those two time points, with a drier period occurring in January, compared to higher average temperatures and higher rainfall in July [35]. Sites sampled in July 2017 are closely clustered except for the site below the Lac de Péligre dam, which is an outlier. This was also the site with lowest diversity relative to other samples collected in July 2017. Since water is released from the bottom of the reservoir, it could be expected that this site would have a different microbial composition compared to the other sampling sites.

### 4.3. Bacterial Diversity

Of all replicates in the January 2018 sampling, the majority of bacteria detected originated from either the Alphaproteobacteria and Gammaproteobacteria classes (Figure 2). From the Gammaproteobacteria class, two bacterial genera of concern that were detected were *Legionella* and *Acinetobacter*. The total relative abundance of the family *Legionellaceae* was a low 1%. In contrast, the total relative abundance of *Acinetobacter* spp. was much higher, 27%, and *Acinetobacter* spp. were detected the most frequently in all five sites from the January 2018 samples, particularly from the Lascahobas samples (Figure S3), though this site also showed the largest variability among replicates (Figure 3). *Acinetobacter* spp. are nosocomial pathogens that survive for extended periods in water, including in drinking water [36], and are associated with multiple antibiotic resistance and a number of clinical outcomes, including pneumonia, wounds, and respiratory and GI tract infections [37,38,39]. Additionally, the species *Acinetobacter baumannii* is implicated in ~80% of hospital acquired *Acinetobacter* infections [40] and in this study comprised 5% of *Acinetobacter* species and 1% of total relative abundance (Figure 2). Again, it should be noted that these relative abundances may have been affected by the failure of the cold chain.

In samples collected in July 2017, two non-toxigenic (environmental) strains of *V. cholerae* were detected (Table 3); the Haitian strain, HE-45, was detected at the La Thème, Lascahobas site and at the site above the Lac de Péligre, and the Chesapeake Bay environmental isolate, RC385, was detected at the Maïssade site. When virulence genes were examined, the *V. cholerae intI1* gene was identified at these sites. The gene *intI1* is included in a class of resistance integrons implicated in the spread of antibiotic resistance via horizontal gene transfer [41]. In the July 2017 sampling sites, *intI1* was not detected in the absence of *V. cholerae*. In contrast, *V. cholerae intl1* gene was detected in almost 90% of samples collected in January 2018, although *V. cholerae* was detected in less than 50% of the samples, and only at the species level.

Although the focus of most prior studies has been on *V. cholerae*, one metagenomic study has been conducted to the southwest of Hinche, primarily in the region of the Rivière Hinquitte, a tributary of the Rivière Guayamouc [42]. This study examined bacterial diversity in source and point-of-use water. Consistent with our data, *Acinetobacter* was a dominant genus. Surprisingly, *Klebsiella* was the most dominant genus found in their study, yet was only present at low relative abundance in our study in unfiltered data (0.03%; 0.01% from the Rivière Guayamouc site). However, *Klebsiella pneumoniae* virulence genes were dominant at most of our sites. The majority of other dominant bacterial genera found in the two river water sources sampled in their study were also present at our sites, but also at low relative abundance (<0.2%). These findings could reflect the proximity of water sources used for drinking to the consumers, and hence to minimal sanitation, whereas our samples were taken at larger river sites with potentially greater input from agricultural and other sources. For example, dominance of *Sphingobium yanoikuyae* (Figure S3), often associated with PAH-contaminated soils [43], at the Mirebalais Rivière La Thème site, as well as the presence of predatory *Bacteriovorax* spp. [44], will change the bacterial community structure in our study compared to the pathogen-dominated genera in the source and point-of-use study [42].

The finding of dominance of *Klebsiella pneumoniae* virulence genes in our samples, in the absence of much evidence for the bacterium itself, speaks to the importance of a complete metagenomic approach for potential health risk assessments from environmental samples. While there is evidence for environmental reservoirs of virulence factors in the absence of clinically important pathogens [45], the presence of these factors should not be ignored due to rapid dissemination through horizontal gene transfer, and further studies in this area are warranted [46].

### 4.4. Phage and Virulence Factor Diversity

Almost all the bacterial pathogens reflected by the presence of phages were detected at low relative abundance in the filtered data set with the exception of *Cronobacter* spp., *Haemophilus* spp. *Shigella* spp., and *Vibrio* spp. *Cronobacter* spp., *Shigella* spp., and *Vibrio* spp., were detected in the unfiltered dataset, but *Haemophilus* spp. was not detected, which is surprising given that the *Haemophilus* phage, HP1 was detected at almost all sites in unfiltered data, and as high as 9.1% relative abundance at one of the Hinche River sites. Important human pathogens reflected by identified virulence factors were also present in the filtered dataset, including *Klebsiella pneumonia*, *Pseudomonas aeruginosa*, and *E. coli*, and in the unfiltered dataset, *V. cholerae*. However, *Proteus mirabilis* was not detected, although this opportunistic pathogen is commonly found in soil and water [47].

### 4.5. Fungal and Protozoan Diversity

Fungi of greatest concern include *Onygenales* spp., which dominated most of the samples and includes a number of emerging human pathogens [48], *Candida parapsilosis* [49], *Enterocytozoon bieneusi* [50], and *Anncaliia algerae* [51]. Unfiltered results also suggest the presence of *Candida albicans*, *Alspergillus fumigates* and *Pneumocystis jirovecii* (the causative organism of *Pneumocystis pneumonia*). All of these pathogenic fungi are of concern, especially in Haiti where HIV infection rates remain very high, and have been shown to be a risk factor in susceptibility to cholera in Haiti [52]. 

Some pathogenic protozoan sequences are also of concern, including sequences representative of *Acanthamoeba polyphaga* at high relative abundance at two sites and the malaria parasite, *Plasmodium falciparum* at almost all sites. Presence of potentially pathogenic *Entamoeba* spp., and *Toxoplasma gondii* in unfiltered data is also of concern. Although not a human pathogen, the finding in unfiltered data of *Trypanosome congolense*, a major cause of African animal trypanosomosis, has implications for a subsistence agricultural economy [53].

### 4.6. Cholera and EHEC Concerns

The cholera toxin (CTX) converting phage is associated with cholera toxin production by *V. cholerae* and was detected in one sample collected at the Thomonde site (Table 4). Detection of the *V. cholerae intI1* gene and the CTX converting phage are indicative of a potential risk for enhanced cholera toxin production by these strains. While waters sampled in this study were not sources used for drinking water, many of them are heavily used for bathing, washing clothes, and for other household activities where water could be inadvertently consumed.

Shiga toxin converting phages were detected at most of the sites (~70%) sampled in January 2018 (Table 3). These phages are important in Shiga toxin production by EHEC, a pathogen that can cause disease with high mortality risk [54]. Although all sites were positive for *E. coli* at low relative abundance during the January 2018 sampling, only one was definitively identified as EHEC from the Lascahobas site at 0.06% relative abundance, where the Shiga toxin-producing EHEC serotype 0157:H7 was also detected. All sites sampled in July 2017 were positive for *E. coli*, at generally higher relative abundance than January samples, with the highest relative abundance of 3.48% at the Lascahobas site, most likely reflecting the relatively higher temperatures and surface runoff from significant rainfall that had occurred over an extended period. 

Predictive models of disease events that combine both environmental parameters with knowledge of the presence of putative pathogens and virulence or antibiotic resistance genes in environmental samples, may be particularly useful in developing preventive strategies against a cholera outbreak. Surveillance efforts, in combination with simple interventions such as bio-sand filters [10] and development of an effective cholera vaccine [55,56,57] will aid in the elimination of this disease. Once optimized, hand-held sequencing technologies could then potentially be used as diagnostic tools and for source-tracking of pathogens. Overall, cholera surveillance requires multiple methods to gather accurate data in a timely manner for decision-making purposes, but this preliminary research provides an initial context for demonstrating the potential use of metagenomics to identify sources of cholera, track cholera-related genes and bacteriophages, and identify bacterial community fingerprints for different areas in Haiti.

## 5. Conclusions

These preliminary results suggest that sequencing DNA from environmental water samples and subsequently applying metagenomic analysis offers a useful approach to characterize environmental samples collected from heavily used water bodies in Haiti. Toxigenic *V. cholerae* O1 and O139 strains were not detected in this analysis, consistent with the recent decline in cholera cases, although environmental *V. cholerae* strains and converting phages for both cholera and Shiga toxins were detected, indicating that a potential disease risk remains for nearby populations. While further sample collection and greater in-depth analysis are needed, the results of this preliminary study provide insight and offer a potential monitoring tool for detecting the re-emergence of toxigenic *V. cholerae* and other waterborne diseases in the aquatic environment in Haiti. 

## Figures and Tables

**Table 1:** Location and description of sampling sites.

Site (Approximate Position on Map)	Coordinates	Description
		January 2018
1. Rio Frio, Maïssade	19.1703 N 72.1353 W	Small tadpoles and fishes were visible in this water. Water depth was 2–3 feet and the water was very turbid.
2. Rivière Guayamouc, Hinche	19.1494 N 72.0092 W	Trash piles and foraging pigs were present on the river banks. The water was extremely turbid.
3. Rivière de Thomonde, Thomonde	19.0082 N 71.9520 W	Many people washed clothes and bathed at this site. The water was turbid and sediment a unique silver gray color.
4. Rivière La Thème, Mirebalais	18.8356 N 72.1071 W	The water was turbid and served as irrigation for the sweet potato field nearby. Algae growth was present on the river banks. Trash bags and other plastics were floating on the sides of the river.
5a. Rivière Lascahobas, Lascahobas	18.8308 N 71.9451 W	The water was clear with a visible rocky bed. The sample was easily filtered. However, the river bank contained numerous plastic waste products.
		July 2017 (samples collected during this time were not replicated)
1. Rio Frio, Maïssade	19.1703 N 72.1353 W	People crossed their vehicles here, bathed, and washed clothes.
3. Rivière de Thomonde, Thomonde	19.0082 N 71.9520 W	The water was clear and this site was upriver of considerable activity, but was also used for bathing and washing clothes.
4. Rivière La Thème, Mirebalais	18.8356 N 72.1071 W	This site contained the most contaminated water comparatively. There was open defecation on the side of the river, trash piles, and many people washing clothes.
5b. Tributary of Rivière la Peigne, Lascahobas	18.8204 N 71.9464 W	Water from this site came from a groundwater spring through a water pipe out of the side of a mountain. Many people were bathing near the pipe, with clothes washing below the sampling point.
6. Rivière Artibonite, below the Lac de Péligre	18.9026 N 72.0604 W	Water was collected about a mile below the dam where it was released at the bottom of the reservoir.
7. Rivière Cabestor, Cabestor	18.8689 N 72.0011 W	Water from the Rivière Cabestor mixed with water from a small creek near the MFH birthing center. At this sampling point, vehicles crossed the river, and people bathed and washed clothes.

**Table 2:** Elevation, average temperature, and average rainfall for January and July at largely populated communities close to the sampling sites [24].

Site Location	Elevation (Meters)	Average Temperature (°C)		Average Precipitation (mm)	
		January 2018	July 2017	January 2018	July 2017
Maïssade	270	22.4	25.8	30.8	151
Hinche	238	21.6	25	28.1	155.2
Thomonde	284	Monthly data not available for this site, ~10 miles from Hinche			
Mirebalais	120	22.2	25.7	23.5	150.3
Lascahobas	219	20.3	23.7	29.5	136.3
Cabestor	300	Monthly data not available on this site, <10 miles from Lascahobas			
Lac de Péligre	175	Monthly data not available for these sites (both for above and below the Lac de Péligre)			

**Table 3:** Relative abundance of sequences characterizing *Escherichia coli*, *V. cholerae*, *V. cholerae Intl1* converting phage and *Stx2* (Shiga toxin gene) converting phage.

Site	Replicate	January 2018			
		*E. coli*	*V. cholerae*	*V. cholerae Intl1*	*Stx2*-converting phage
Maïssade	1	0.12	ND	1.61	ND
	2	0.01	<0.01	4.91	8.45
	3	<0.01	ND	ND	0.70
Hinche	1	0.04	ND	2.69	0.22
	2	<0.01	<0.01	4.8	ND
	3	<0.01	ND	0.11	ND
Thomonde	1	0.03	<0.01	6.93	ND
	2	0.01	<0.01	6.05	0.52
	3	0.03	0.02	3.52	7.11
Mirebalais	1	<0.01	ND	7.59	4.31
	2	<0.01	ND	18.45	9.82
	3	0.01	<0.01	7.55	7.62
Lascahobas	1	0.19	<0.01	2.13	1.78
	2	0.06 *	ND	6.40	0.11
	3	0.02	ND	ND	0.36
		**July 2017**			
Maïssade	1	1.11	<0.01 **	17.65	ND
Mirebalais	1	0.16	<0.01 ***	7.18	ND
Lascahobas	1	3.57	<0.01 ***	3.09	0.77
Below Péligre	1	0.14	ND	ND	ND
Cabestor	1	0.34	ND	ND	ND
Above Péligre	1	0.08	<0.01 ***	3.31	ND

Notes: * *E. coli* O157:H7; ** *V. cholerae* RC385; *** *V. cholerae* HE-45; ND—not detected. All data are presented as percentages.

**Table 4:** Selection of some of the more dominant viruses and virulence factors detected in samples collected at the Thomonde site, replicate #3 from January 2018 sampling (filtered data, * indicates *V. cholerae* virulence factors from unfiltered data that require further confirmation). All classes of AMR genes detected in filtered data from this sample are also reported.

Viruses and Bacteriophages	Virulence Associated Genes	Classes of AMR Genes Detected
*Enterobacteria* phage HK630	*Klebsiella pneumoniae: orf6, GI 42543951, tnpA*	Aminoglycosides: *aadA*, *aadA5, aadA7, aadA16, aadA10, aac1, aac3 Ia, aph6 Id, aph, ant2’’ Ia*
Siphoviridae_u_s	*Enterobacter aerogenes: traM, tniB, ssb, korC*	Sulphonamide sul2
*Escherichia* virus P1	*V. cholerae: intI*1, GI 42567126 *, VCA0118 *, CARB-6 *	Trimethoprim: *dfrC*
Viruses_u_s	*Serratia marcescens*: *orfA, intI3*	Beta-lactam: *bla*OXA, *bla*AIM
*Stx2*-converting phage 1717	*E. coli: qacEdelta1, aphA7*	Macrolide: *mphE*, *ermA*
Human mastadenovirus C	*Pseudomonas aeruginosa: intI*1, *accC*1, *aadA*6	Phenicol: *dha*1
*Enterobacteria* phage BP-4795	*Salmonella Infantis: tnpR*	
G7cvirus_u_s	*Pseudomonas putida: qacEdelta*1	
Myoviridae_u_s	*Proteus mirabilis*: *sul*1	
*Vibrio* phage CTX	*Morganella morganii: oxa-*2	

## Supplementary Materials

The following are available online at http://www.mdpi.com/1660-4601/15/10/2211/s1, Figure S1. Mean Sequence Quality distribution representing the number of reads with average quality scores. Figure S2. Sequence Quality Histogram representing the mean quality value across each base position in the read. Figure S3. Top 11–13 most abundant (relative) bacteria for each replicate at the (a) Maïssade, (b) Hinche, (c) Thomonde, (d) Mirebalais, and (e) Lascahobas sites for January 2018 sampling. Figure S4. Filtered data for Viruses. Figure S5. Filtered data for top 80% virulence factors. Figure S6. Filtered data for top 50% AMR. Figure S7. Filtered data for Fungi. Figure S8. Filtered data for Protists. Table S1. DNA concentrations and general sequencing statistics of January 2018 samples measured by MultiQC. Table S2. Choa1 alpha diversity raw data used to generate the box plots for Figure 3.
