# Supplementary material for: A Metagenomic Approach to Evaluating Surface Water Quality in Haiti
Source: Int J Environ Res Public Health. 2018 Oct 10;15(10):2211. doi: 10.3390/ijerph15102211 (PMC6209974; doi:10.3390/ijerph15102211)
Supplement: Supplementary file 1 [file ijerph-15-02211-s001.pdf]

International Journal of
Environmental Research and Public Health

Supplementary Materials

A Metagenomic Approach to Evaluating Surface Water Quality in Haiti

Monika A. Roy, Jean M. Arnaud, Paul M. Jasmin, Steve Hamner, Nur A. Hasan, Rita R. Colwell and Timothy E. Ford

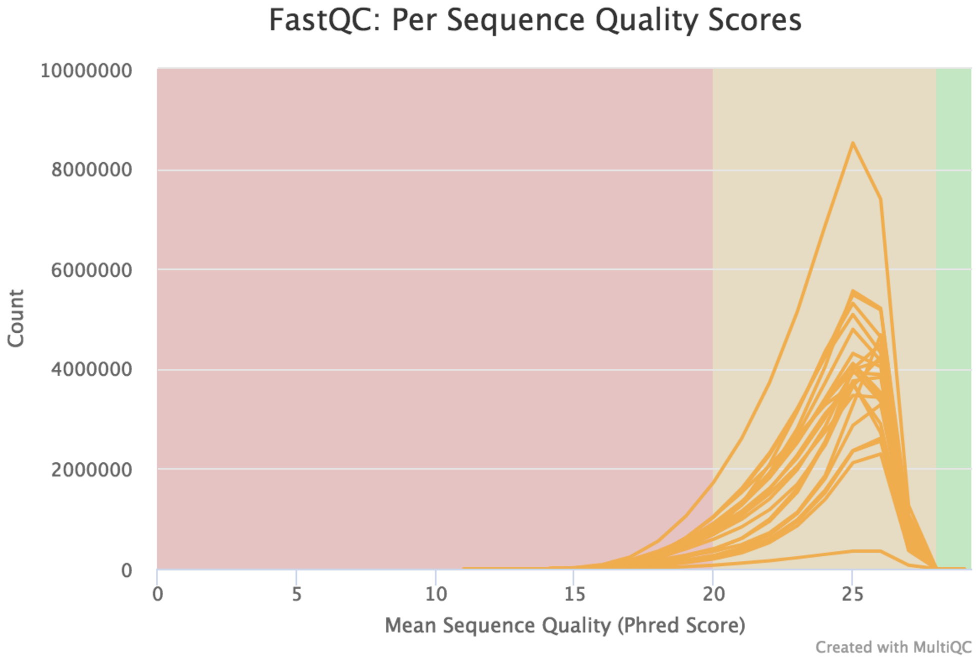

Figure S1. Mean Sequence Quality distribution representing the number of reads with average quality scores.

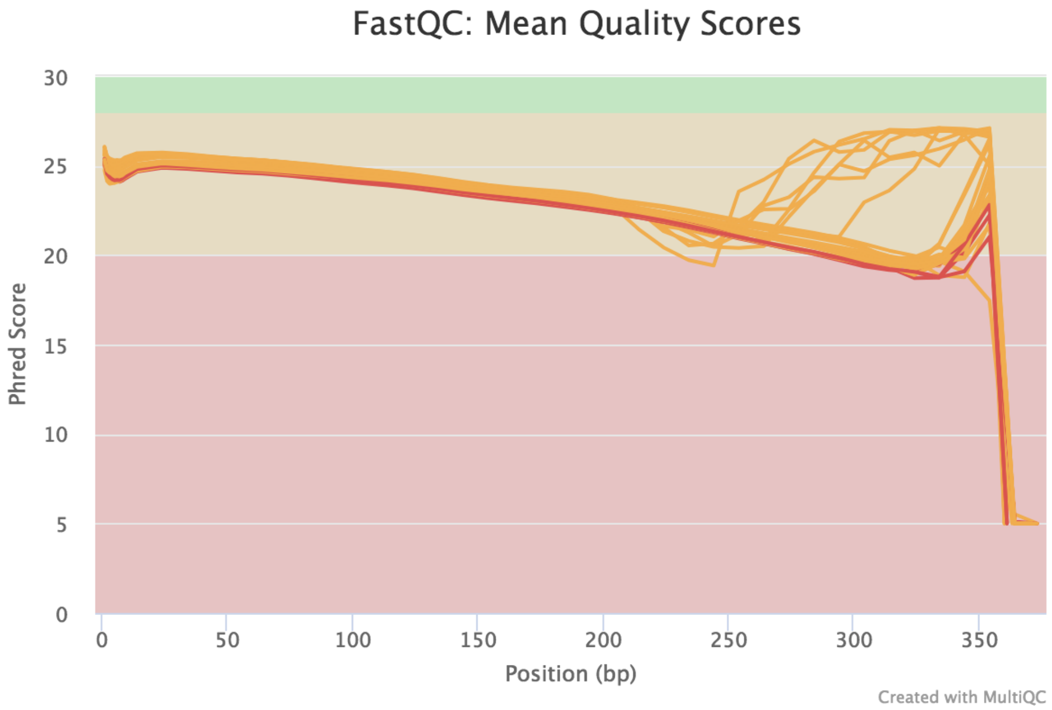

Figure S2. Sequence Quality Histogram representing the mean quality value across each base position in the read.

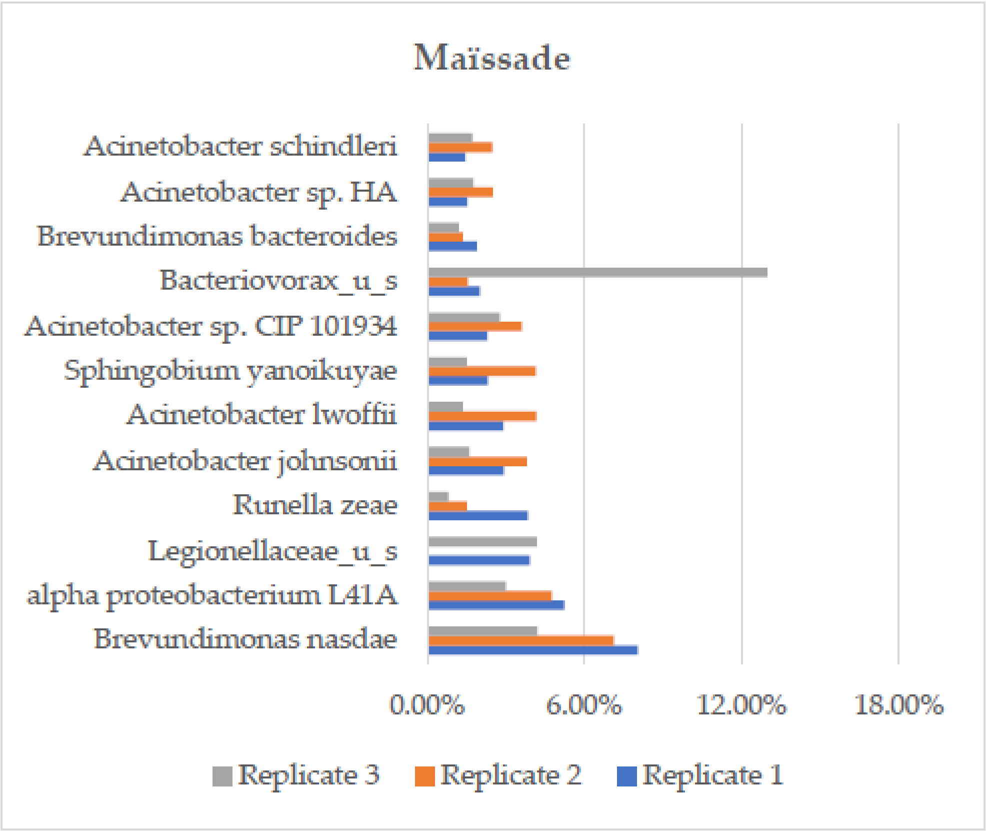

(a)

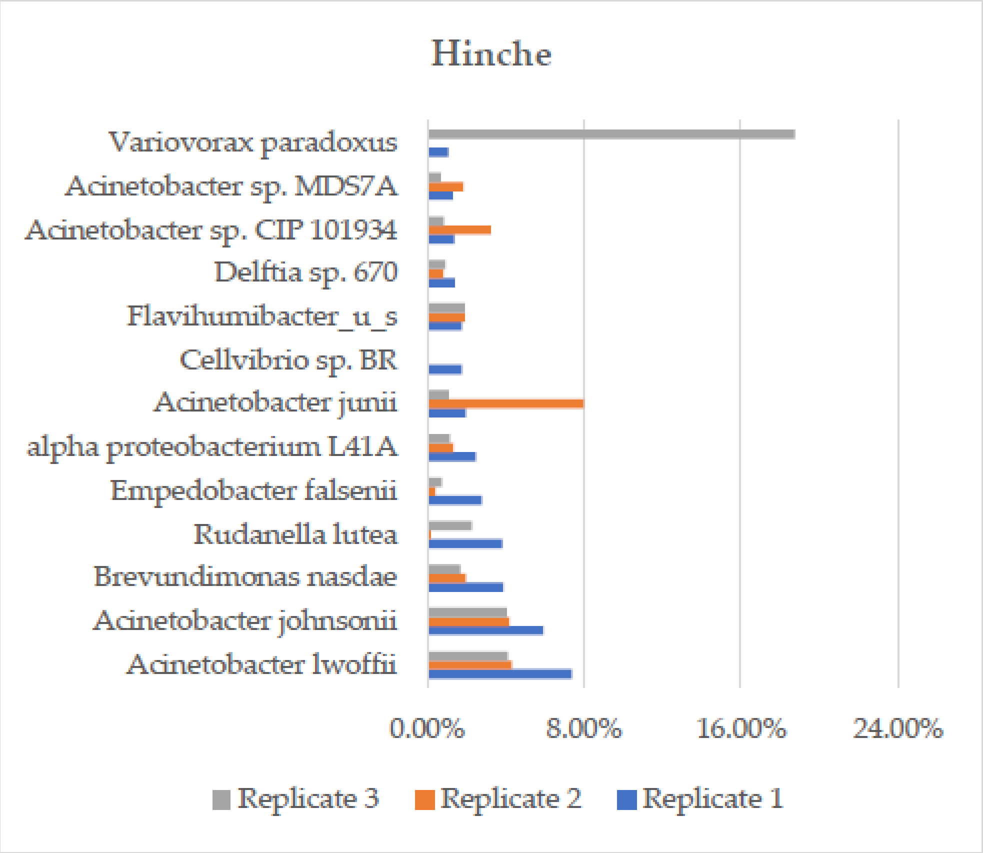
Hinche
Variovorax paradoxus
Acinetobacter sp. MDS7A
Acinetobacter sp. CIP 101934
Delftia sp. 670
Flavihumibacter_u_s
Cellvibrio sp. BR
Acinetobacter junii
alpha proteobacterium L41A
Empedobacter falsenii
Rudanella lutea
Brevundimonas nasdae
Acinetobacter johnsonii
Acinetobacter lwoffii
0.00%
8.00%
16.00%
24.00%
Replicate 3
Replicate 2
Replicate 1

(b)

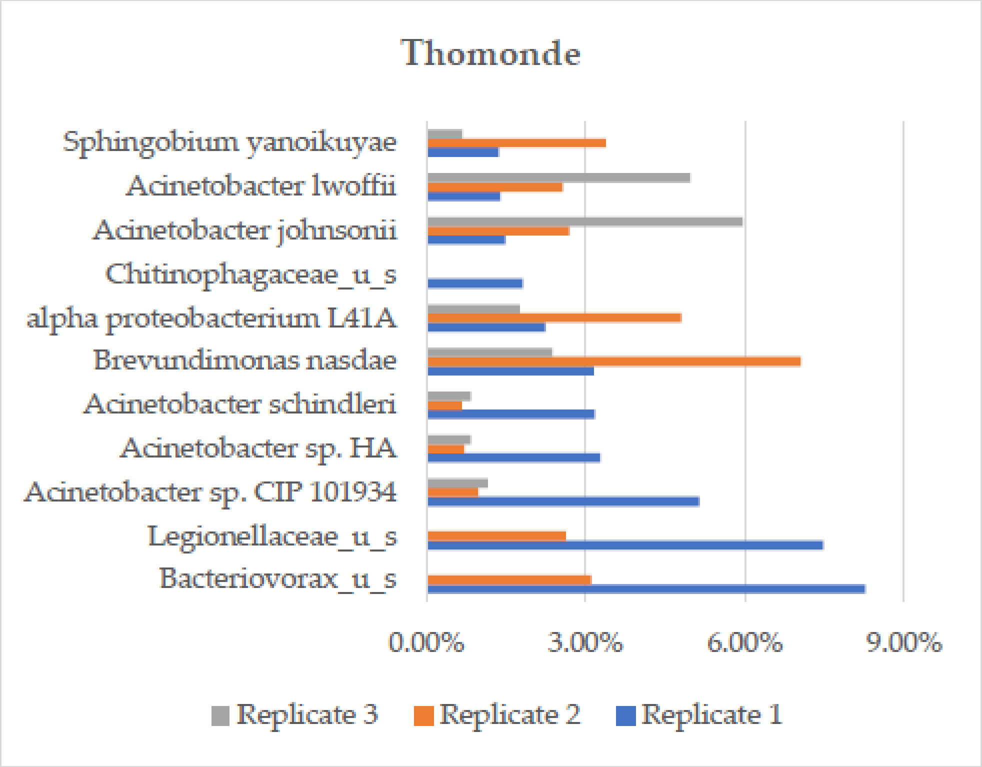
Thomonde
Sphingobium yanoikuyae
Acinetobacter lwoffii
Acinetobacter johnsonii
Chitinophagaceae_u_s
alpha proteobacterium L41A
Brevundimonas nasdae
Acinetobacter schindleri
Acinetobacter sp. HA
Acinetobacter sp. CIP 101934
Legionellaceae_u_s
Bacteriovorax_u_s
0.00%
3.00%
6.00%
9.00%
Replicate 3
Replicate 2
Replicate 1

(c)

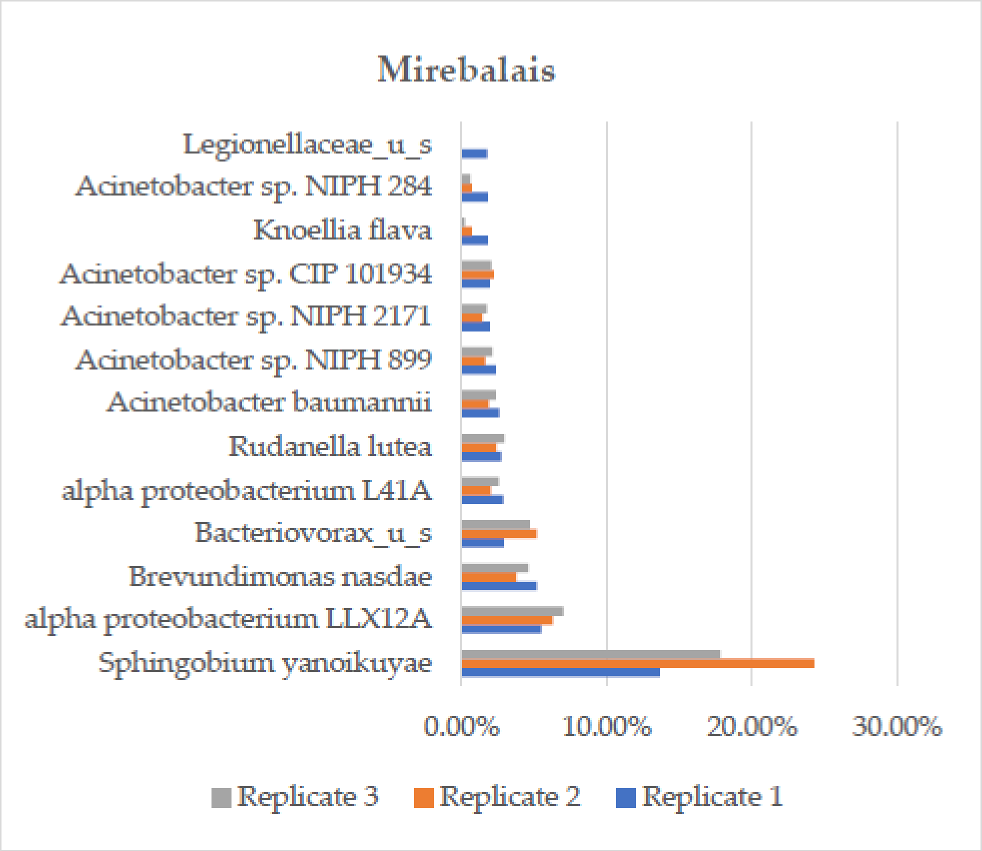

(d)

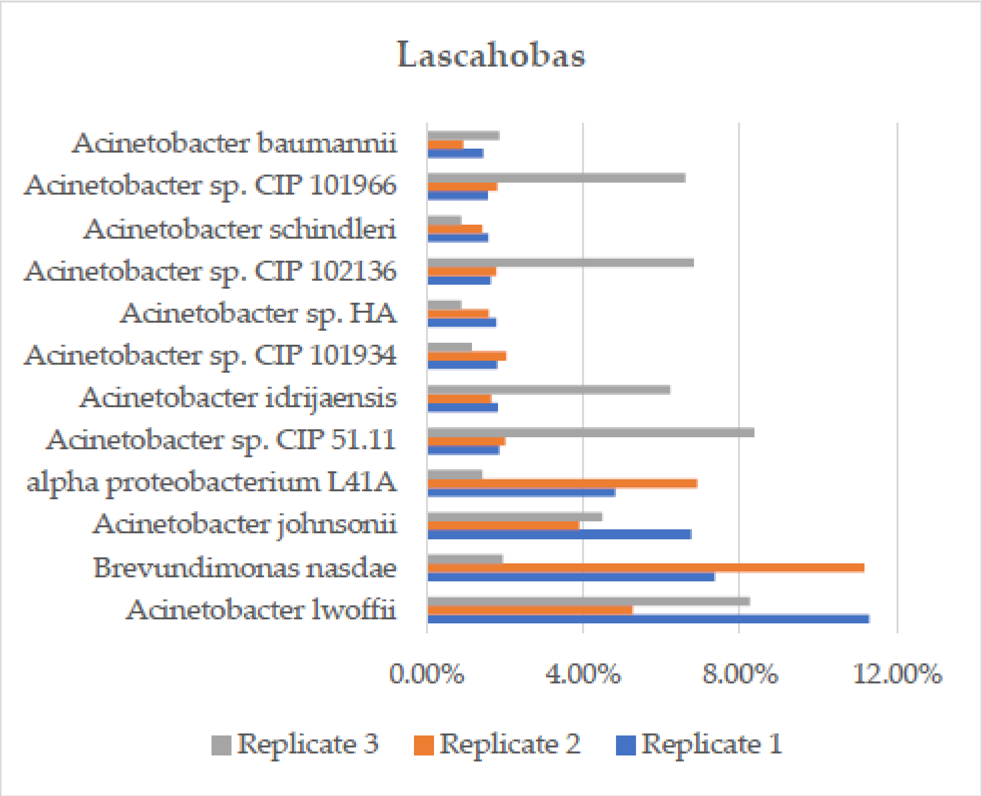

(e)

Figure S3. Top 11–13 most abundant (relative) bacteria for each replicate at the (**a**) Maïssade, (**b**) Hinche, (**c**) Thomonde, (**d**) Mirebalais, and (**e**) Lascahobas sites for January 2018 sampling.

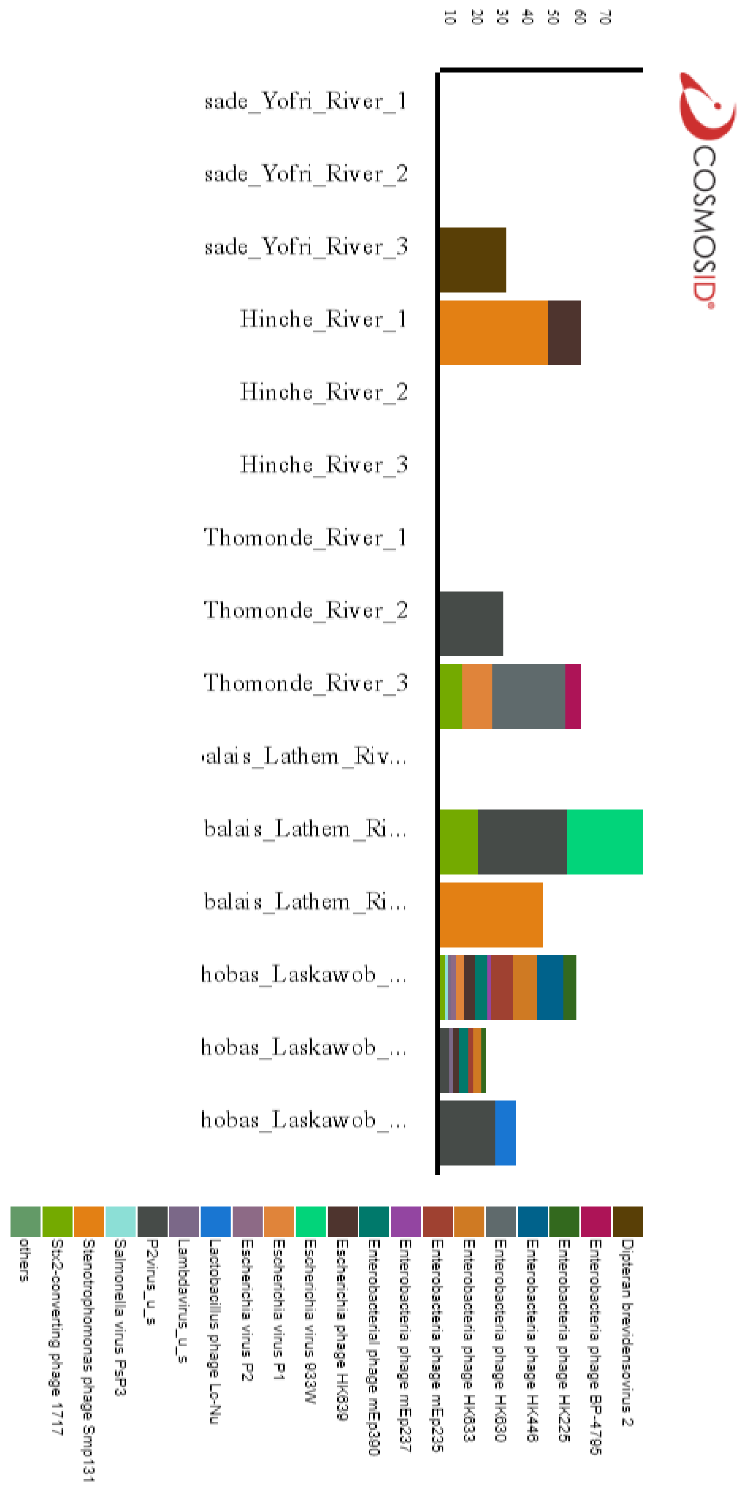

Figure S4. Filtered data for Viruses.

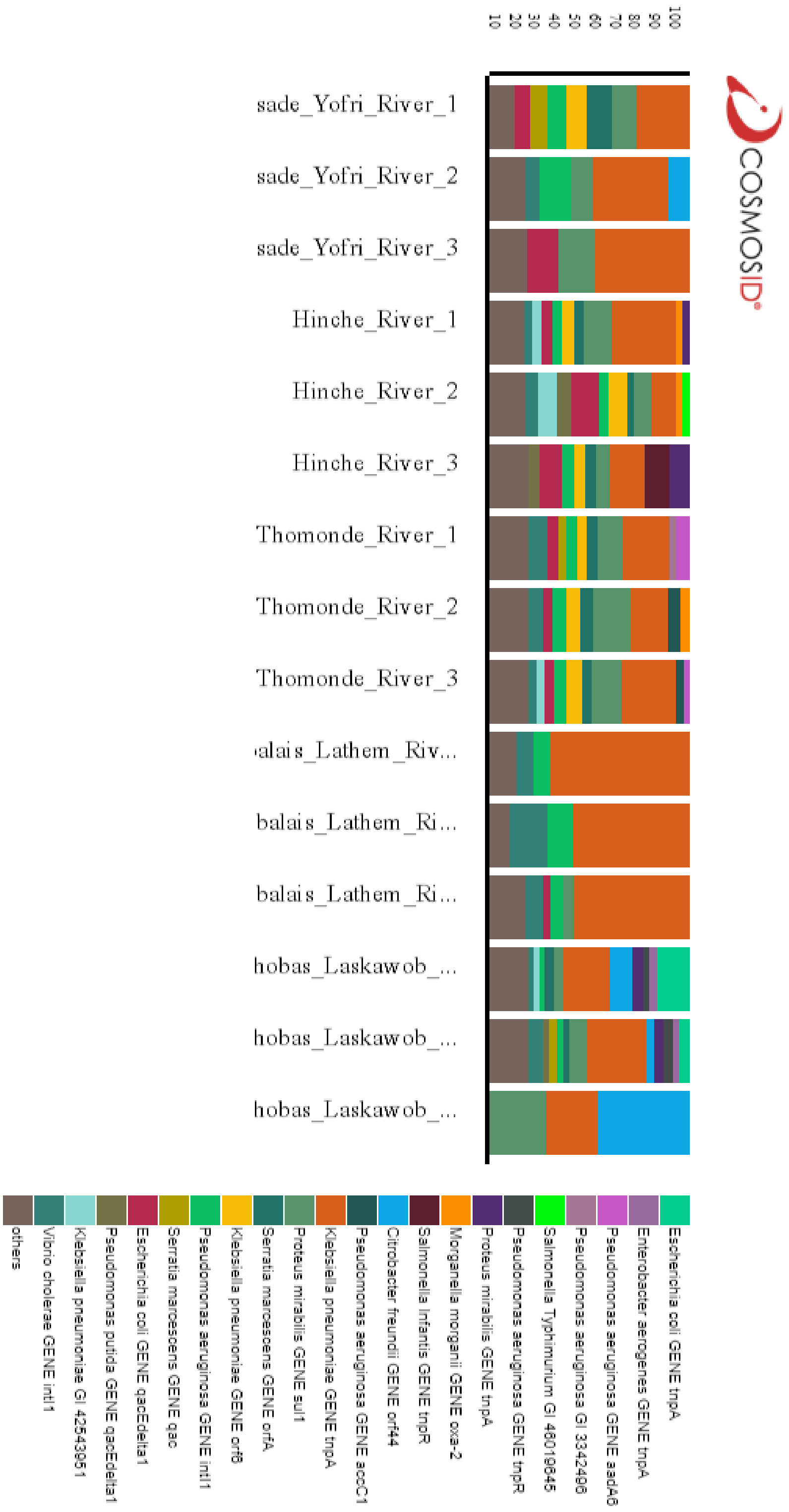

Figure S5. Filtered data for top 80% virulence factors.

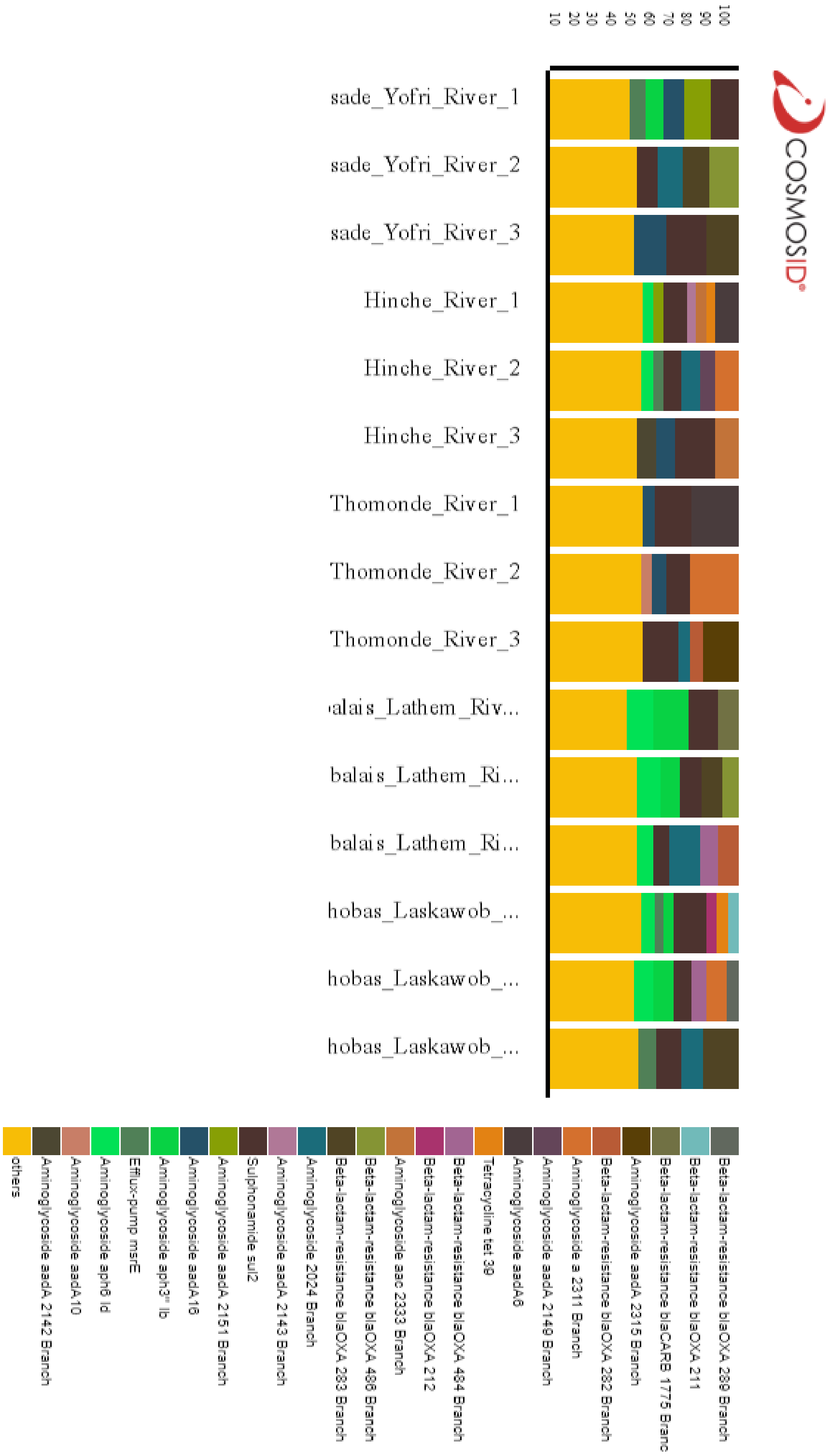

Figure S6. Filtered data for top 50% AMR. AMR: antimicrobial resistance.

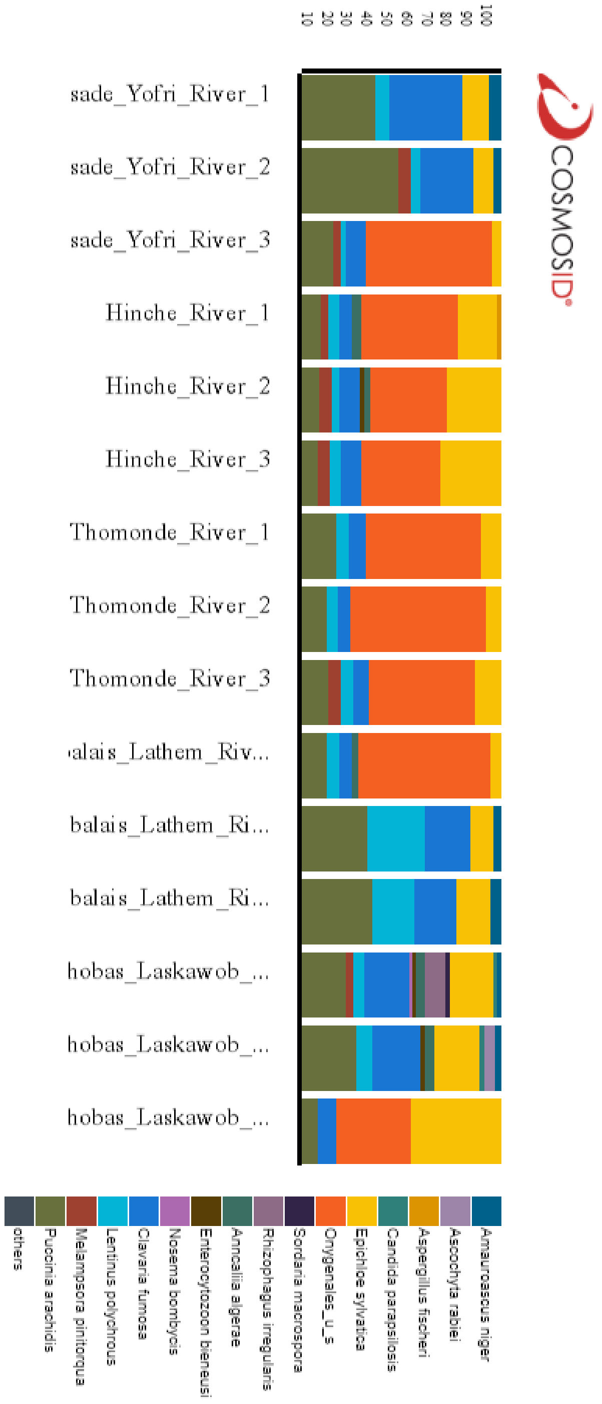

Figure S7. Filtered data for Fungi.

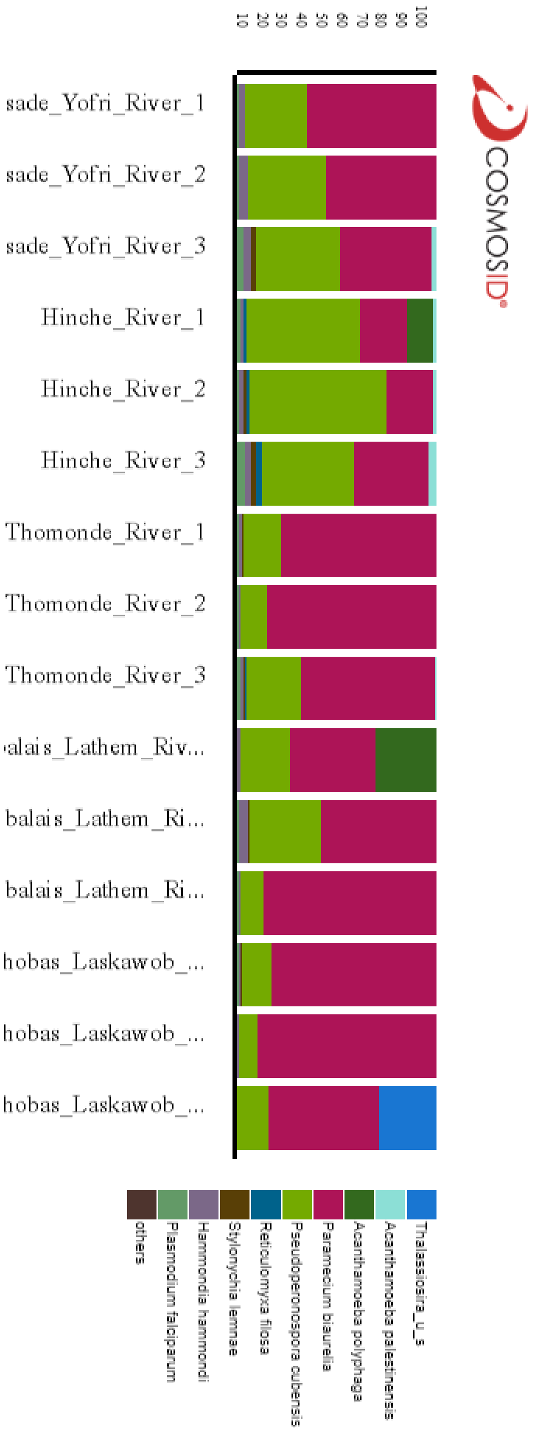

Figure S8. Filtered data for Protists.

Table S1. DNA concentrations and general sequencing statistics of January 2018 samples measured by MultiQC.

Samples	Replicate	DNA Concentration (ng/µL)	% Dups	% GC	Length	M Seqs
Maïssade	1	1.5	9.50%	59%	157 bp	18.40
	2	1.29	9.80%	60%	171 bp	18.80
	3	0.13	10.10%	57%	178 bp	18.70
Hinche	1	2.5	14.50%	56%	155 bp	23.80
	2	4.1	18.80%	54%	161 bp	21.10
	3	2.14	18.70%	53%	164 bp	16.30
Thomonde	1	3.48	9.40%	57%	168 bp	19.30
	2	2.12	9.20%	57%	141 bp	16.30
	3	1.83	9.80%	55%	166 bp	20.00
Mirebalais	1	3.88	9.60%	61%	154 bp	16.70
	2	2.52	10.30%	58%	175 bp	23.60
	3	2.5	10.40%	57%	174 bp	18.90
Lascahobas	1	3.36	16.20%	54%	167 bp	39.20
	2	1.71	14.60%	55%	160 bp	25.10
	3	2.12	52.70%	50%	145 bp	17.90

Table S2. Choa1 alpha diversity raw data used to generate the box plots for Figure 3.

Sampling Site and Replicate	CHOA1
Hinche_River_1	1230.93
Hinche_River_2	1157.46
Hinche_River_3	888.89
Lascahobas_Laskawob_River_1	1443.57
Lascahobas_Laskawob_River_2	1179.08
Lascahobas_Laskawob_River_3	606.74
Maissade_Yofri_River_1	908.7
Maissade_Yofri_River_2	1028.44
Maissade_Yofri_River_3	980.58
Mirebalais_Lathem_River_1	920.69
Mirebalais_Lathem_River_2	1147.68
Mirebalais_Lathem_River_3	980.28
Thomonde_River_1	1053.65
Thomonde_River_2	893.96
Thomonde_River_3	1111.88
